# Observations on the Influence of Tropical Climates on Consumptive Habits, Chiefly with Reference to the Seamen, &c. of His Majesty's Navy

**Published:** 1831-03

**Authors:** James Wallace

**Affiliations:** Assistant Surgeon, R.N.


### CONSUMPTION IN TROPICAL CLIMATES.

*Observations on the Influence of Tropical Climates on Consumptive Habits, chiefly with reference to the Seamen, &c. of his Majesty's Navy.* By James Wallace, Assistant Surgeon, R.N.

Although a few may still be doubtful as to the propriety of removing patients threatened with phthisis to a warmer climate, yet, I believe, by far the greater number of medical men are satisfied that such a removal may, under certain circumstances, be attended with the most beneficial results. That it is only at a certain stage of the disease such a removal can be useful, and that it is sometimes difficult to tell how long this stage actually continues, is readily conceded; but that we can, at least, *frequently* point out the period when the chances are in favor of a removal, and that the instances are numerous in proof of this, is no less strenuously maintained. The object of the present paper scarcely renders it incumbent on me to inquire into the causes which produce the favorable change: it may be, alone, the increased cuticular discharge relieving the lungs, or other causes, in conjunction with that, which are to us as yet not very accurately known; but if we are certain of the fact itself, that relief is in some way obtained, it is, perhaps, enough for all practical purposes. I would merely observe, however, that it appears to me to be not only the superior, but also the steady, degree of temperature which is productive of the benefit to consumptive patients in these climates. The prevalency of consumption in England is, I apprehend, not so much to be attributed to the actual degree of cold that we experience, as to the frequent alternations of temperature, by which the circulatory system, and especially that in the lungs, is at one time below par, and consequently at another, from reaction, above it. It is these frequent changes, no doubt, which give origin to the disease in all extra-tropical latitudes; and it is by avoiding these, at an early period, that we are likely to remove, or ward off, this very fatal malady.

It is, perhaps, scarcely possible to tell how late in the disease this remedy can be advised, with any prospect of success; but, as a general rule, I would say, that the sooner the removal is effected, after phthisis is threatened, the better But although the disease has existed for a time, if suppuration has not actually ensued, and the patient's strength remains tolerably good, I think the chances are still in favor of a removal. Even when there has been acute pain in the chest, along with quickened pulse, cough, and other inflammatory symptoms, yet, if the requisite treatment has at once been pursued, and the inflammation has for the time been resolved, then, by removing the patient at once to a tropical climate, we, in all probability, prevent any after attack of pulmonic excitement, and so effectually break the essential link in the morbid chain. Even admitting a second attack of inflammation to ensue ere the patient can be removed, yet, if we can also combat this, so as to prevent the formation of matter, we have still, as before, considerable hope from the remedy. In fact, until we have reason to suppose that the patient is positively expectorating pus, we are justified in recommending the remedy: although, certainly, the longer he waits after phthisis is threatened, the less is his chance of recovery; and, undoubtedly, no time is so eligible for the removal as when the strength is as yet but little impaired, and when, perhaps, slight dyspnœa and cough are almost the only phthisical symptoms.

These remarks have reference chiefly to the strumous or tubercular phthisis, a disease which generally attacks insidiously, and does not arrive, until after a certain period, at suppuration. In these cases, where warning is, as it were, given of the approach of the distemper, the remedy alluded to will, no doubt, be most available; but even in that degree of pulmonary irritation which supervenes on acute inflammation, in robust and naturally healthy subjects, it may still be brought into use with considerable prospect of success; that is, if those hectic symptoms, &c. are absent, which indicate confirmed and irremediable diseased structure. When an acute attack of pneumonia terminates in suppuration, the death of the patient is not only almost certain, but it follows often in an exceedingly short space of time, and scarcely any thing that we know of yet will even retard the fatal issue. But it not unfrequently happens that, although suppuration does not ensue, the inflammation is not altogether resolved, but such a degree of it is retained as gives, at least, a *liability* to suppuration; a state in which an increased and vitiated quantity of mucus is thrown into the bronchiæ, producing cough, difficulty of breathing, &c.; and, in such a state, if the greatest care be not taken, (that is, if there be any additional excitement given,) suppuration is almost immediately induced, and death very speedily follows. In these cases, as well as in threatened tubercular phthisis, I am convinced that a timely removal to a more temperate climate would, in many instances, remove the irritation, and ultimately restore the patient to health.

These few observations, however, are merely introductory to the main object of the paper, which is to recommend that the seamen, &c. of his Majesty's navy, who may have the misfortune to become consumptive, should have the benefit of the remedy adverted to. It is a circumstance well known, that a large number, annually, of the seamen employed in the home station (from the vicissitudes to which they are exposed,) become the subjects of pulmonary disease. These men are sent to hospital as soon as their malady requires it, where they have, no doubt, the benefit of the most skilful advice, with all the other comforts which their unfortunate situation requires; but the majority of them nevertheless die, merely because they are retained in a climate which is altogether hostile to the disease under which they labour. Their disease may thus be alleviated, and their passage to the grave made easy; but their recovery is almost forbidden, because they exist in a temperature which is by far too low, and by far too variable, for the consumptive habit. I have, therefore, imagined that, instead of keeping these patients, indiscriminately, in the hospitals in England, if a selection of the most likely cases were made, and then immediately transmitted to warmer latitudes, a large proportion of them might recover. There are vessels of war, or at least government vessels, proceeding almost every week to southern latitudes, and advantage could easily be taken of them to ship, at each port, those patients that might be deemed eligible for the removal; but, were the plan to be found advantageous, and were patients to become numerous, (as they would certainly do during war,) vessels might be allotted for the express purpose of carrying such invalids to their proper stations. I do not conceive it would matter much where they were sent to, so that they were sent within the tropics: they might be sent to the west coast of Africa, to Fernando Po, Ascension, or any of the West-Indian islands. It is a fact, I believe, pretty well established, that those people who have a tendency to pulmonary complaints are infinitely less liable to the usual diseases of tropical climates than the healthy and robust, and therefore might we send such patients, almost without apprehension, to any, even the most unhealthy situations. If they were removed to a climate adequately and steadily warm, that would be sufficient; and if, by such a removal, namely, to a warm (but, to the robust constitution, an unhealthy) climate, those individuals were to be restored to health, then, in such a climate, they would become valuable men: men who would fill up the blanks produced by the deaths that necessarily occur amongst the robust who arrive from England. At all events, I should think the matter well deserving of a trial; for the probabilities, at least, are in favor of its being advantageous; and, as it is a thing which could be done without any additional expense to government, there could be no objection to it on that head. Such persons are retained, at the public expense, until recovery or death, in the hospitals in England; and at the same expense might they be kept on board our ships at sea. Even admitting that the scheme failed, that the same number died abroad as at home, there would still be something due to us on the score of humanity; but, if a proportion lived, then, in addition to the blessing conferred on the individuals, his Majesty's service would be benefited.

What has now been said with reference to seamen on the home station, is also applicable to some of our naval stations abroad. The Mediterranean has its winter, with considerable vicissitudes of temperature, and pulmonary disease is in that situation also common and fatal. Instead, therefore, of retaining consumptive patients there in the ships, or in the hospital at Malta, or remitting them to the hospitals in England, they might be sent, by the earliest opportunity, to any of the before-mentioned places. The crews of the vessels stationed at the Cape of Good Hope also experience, at a particular season, very inclement weather, and such individuals as required the change might be removed, when convenient, farther northward, either in the Indian sea, or on the westward of the African continent. The vessels, too, towards the northern and southern extremes of America also experience cold in a very great degree, with very frequent alternations of temperature, and the sick from these might, as circumstances required, be removed to a more convenient situation. In short, on most of our naval stations, patients attacked with pulmonary disease could be easily and rapidly removed to an intra-tropical situation.

It may be said by some, that individuals predisposed to pulmonic affection ought not to be received into the navy, because such individuals can never take part in laborious service without suffering from it, and thereby becoming useless as effective seamen. This is true: a man presenting himself for admission into the navy, with an evident tendency to phthisis, or with decided strumous symptoms of one kind or other, is an ineligible subject; he is incompetent to perform the duties of an able and active seaman, and must therefore be rejected. But as we cannot invariably detect the latent liability to phthisis, and as struma, in all its shapes, is not unfrequently developed, from particular causes, even late in life; and, besides, as all are more or less liable to thoracic inflammation and its consequences, we must always expect, even under the most strict system of examination, to find a portion of our seamen afflicted with these ailments. In war especially, when we are anxious to obtain seamen, even of any description, the quantity of thoracic disease must be very great indeed.

These are merely a few imperfect hints which I have thrown out on the subject, in the hope that others, who are more capable of examining the matter, may direct their attention to it; and, if the scheme should appear plausible, urge it further on public notice: but, in conclusion, I think it necessary to repeat what I have said in the beginning, that I am far from advising the remedy, except in the early stage of the disease, that is, previous to suppuration; for, after this, although the ailment may be palliated, yet death must sooner or later ensue, and, under such unhappy circumstances, the comforts of home must always be preferred to the slight benefit to be derived from a foreign atmosphere.

*H. M. Ship Gloucester; August*, 1830.

